# A Case Report on the Use of a Novel Technique for Intra-operative Nasojejunal Tube Placement in a Patient Undergoing Ivor-Lewis Esophagectomy

**DOI:** 10.7759/cureus.64949

**Published:** 2024-07-19

**Authors:** Fatima Tu Zahara, Ibtissam Bin Khalid, Shahid Khattak, Aamir Ali Syed

**Affiliations:** 1 Department of Surgical Oncology, Shaukat Khanum Memorial Cancer Hospital and Research Centre, Lahore, PAK

**Keywords:** novel technique, case report, enteral feeding, nasojejunal tube, ivor-lewis esophagectomy

## Abstract

Esophagectomy is an important cornerstone in the management of esophageal cancer. Post-operative feeding options in Ivor-Lewis esophagectomy include nasojejunal tube (NJT), feeding jejunostomy, and direct oral feeding. NJT is traditionally placed endoscopically or under fluoroscopic guidance. In this case report we present an alternate technique for NJT placement.

A 55-year-old male presented to our clinic with dysphagia. On esophagogastroduodenoscopy, a gastroesophageal junction (GOJ) tumor was noted. A diagnosis of moderately differentiated adenocarcinoma was made on biopsy. The patient received eight cycles of epirubicin, cisplatin, and capecitabine (ECX), following which an Ivor-Lewis esophagectomy was carried out. This case report highlights the technical aspects and potential pitfalls of placing NJT in patients undergoing Ivor-Lewis esophagectomy without the use of endoscopy or fluoroscopic guidance.

Direct oral feeding after Ivor-Lewis esophagectomy may lead to suboptimal caloric provision while feeding jejunostomy is associated with complications such as dermatitis, wound infection, and intestinal obstruction. On the other hand, endoscopic or fluoroscopic insertion of NJT can expose the anastomosis to potentially harmful mechanical forces. NJT can be easily placed using our technique in patients undergoing hybrid Ivor-Lewis esophagectomy. The safety of this technique can be investigated by further studies.

## Introduction

Esophageal cancer is the seventh most common malignancy worldwide and the sixth most common cause of cancer-related mortality [1]. Advances in the management of esophageal carcinoma have resulted in a significant improvement in overall survival. In spite of these advances, surgery remains an important cornerstone in the management of esophageal cancer.

Depending on the level of esophagus involved in the disease, possible surgical options include Mckewon esophagectomy and Ivor-Lewis esophagectomy [2]. Both of these are complex surgical procedures with considerable associated morbidity. Most of the patients with esophageal cancer are malnourished because of disease-related dysphagia. Therefore, the role of nutritional support in pre-operative rehabilitation and the post-operative period is of paramount importance. Early post-operative enteral feeding is associated with reduced post-operative complication rates and a shorter duration of hospital stay [3]. Post-operative feeding options include nasojejunal tube (NJT), feeding jejunostomy, and direct oral feeding. The ideal route of administration of enteral feeding is still an area of controversy [4].

The objective of the present report is to describe a surgical technique for NJT placement in Ivor-Lewis esophagectomy without the use of an endoscope. This case report has been reported in line with SCARE2023 criteria [5].

## Case presentation

A 55-year-old male presented at our institution with a complaint of dysphagia. The patient had no comorbidities or family history of malignancy. On esophagogastroduodenoscopy, a plaque-like stenotic tumor was seen, starting at 37 cm from the incisors and extending distally to involve the gastroesophageal junction (GOJ). The tumor involved cardia circumferentially. On biopsy, a diagnosis of moderately differentiated adenocarcinoma was made. There was no evidence of distant metastasis and on staging laparoscopy, the tumor was noted at GOJ, cardia, and proximal lesser curve of the stomach. A multidisciplinary team recommended peri-operative chemotherapy.

The patient received four cycles of epirubicin, cisplatin, and capecitabine (ECX). On completion of these, a re-staging CT scan showed a poor response to chemotherapy and it was, therefore, decided to administer the remaining four cycles of ECX as well. Re-staging laparoscopy revealed a stable tumor at GOJ, cardia, and proximal lesser curvature of the stomach. Given these findings and in accordance with institutional practices, we decided to carry out a hybrid Ivor-Lewis esophagectomy.

With the patient in the lithotomy position, laparoscopic gastric mobilization was carried out. After complete mobilization of the stomach, a 12 Fr NJT was inserted by the anesthesia team and was guided across the pylorus laparoscopically. Partial preparation of gastric conduit was carried out using an endostapler, in such a way that the conduit remains attached to the specimen with a bridge of gastric tissue at the greater curvature. Care was taken to avoid accidental transaction of NJT while using an endostapler and the tube was directed toward the greater curvature so that it passed across the previously mentioned bridge of gastric tissue. The partially prepared conduit with NJT passing through it was tucked at the hiatus, following which the position of the patient was changed to left lateral.

**Figure 1:**
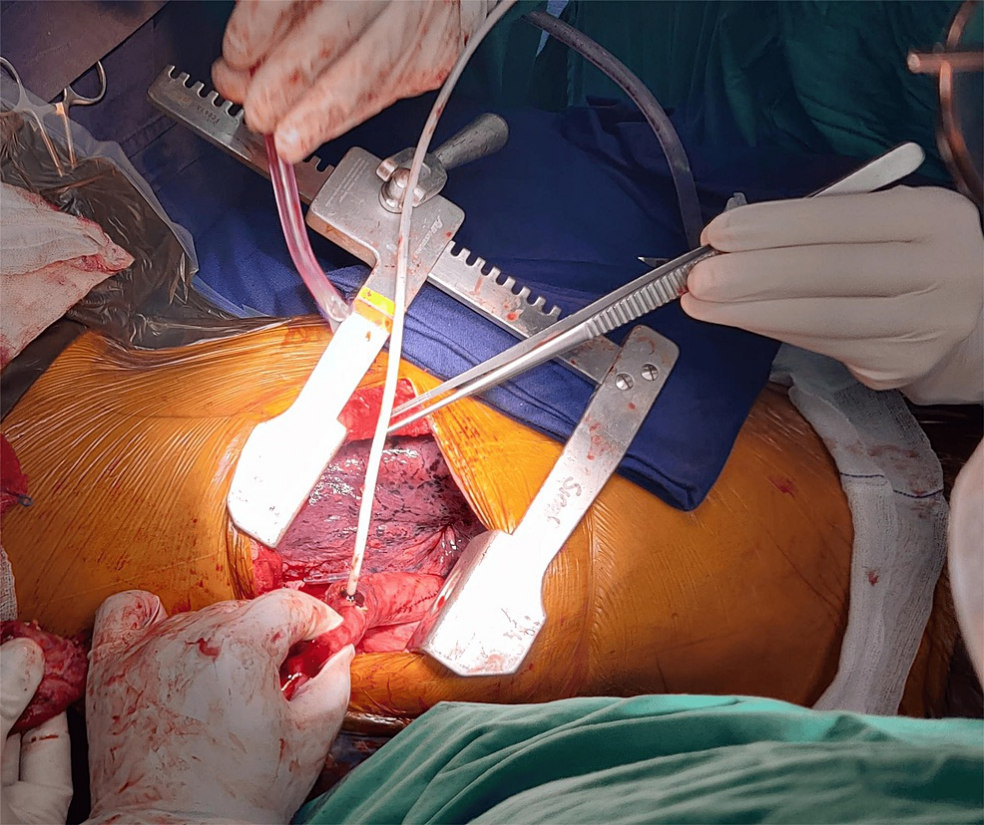
An opening is created on the side of the conduit at the site of the future anastomosis to deliver out the proximal part of the NJT so that an endostapler may be used to carry out distal transection. NJT, nasojejunal tube

**Figure 2:**
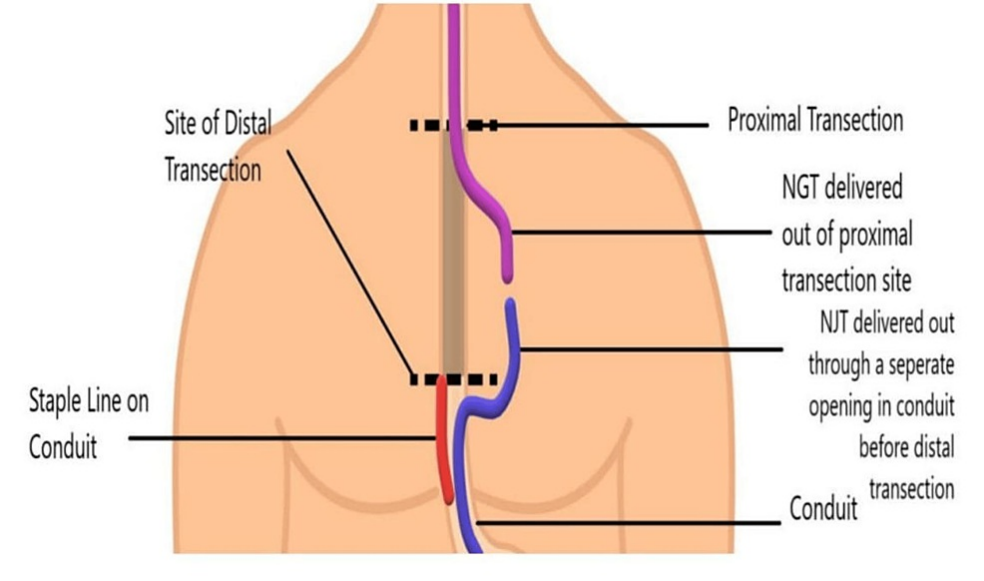
After being delivered through the side of the conduit, NJT is sutured to the NGT. The figure is the author’s own creation. NGT, nasogastric tube; NJT, nasojejunal tube

**Figure 3:**
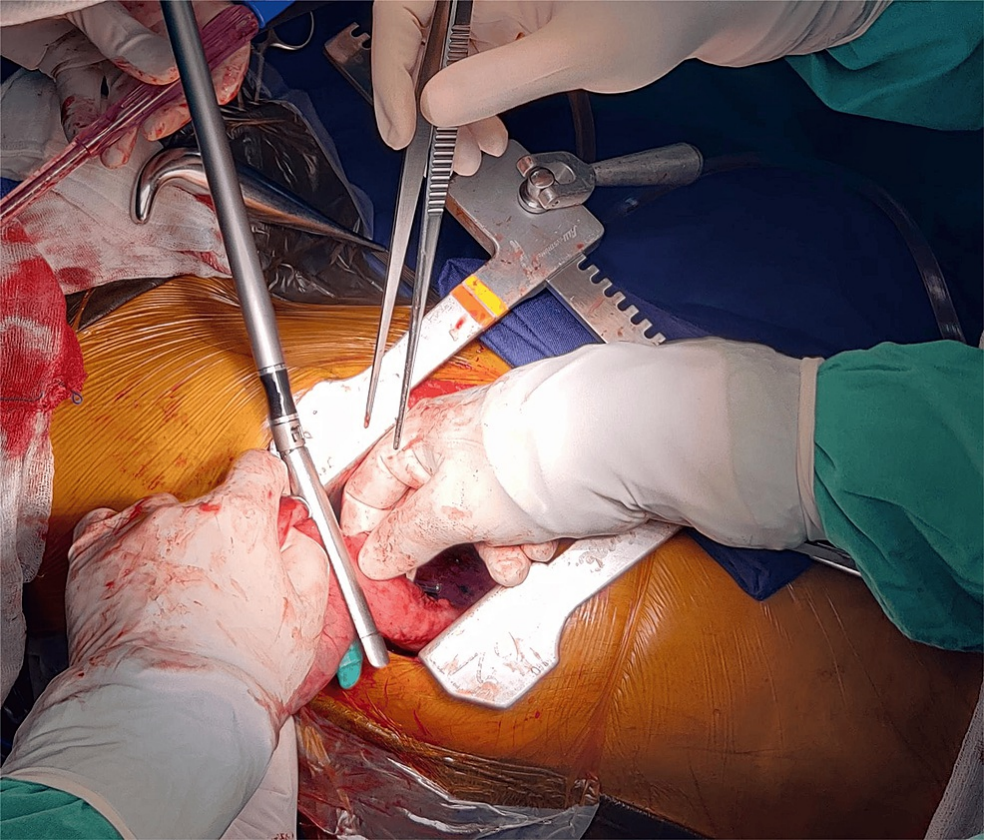
Endostapler is used to complete distal transection, thereby separating conduit and specimen.

**Figure 4:**
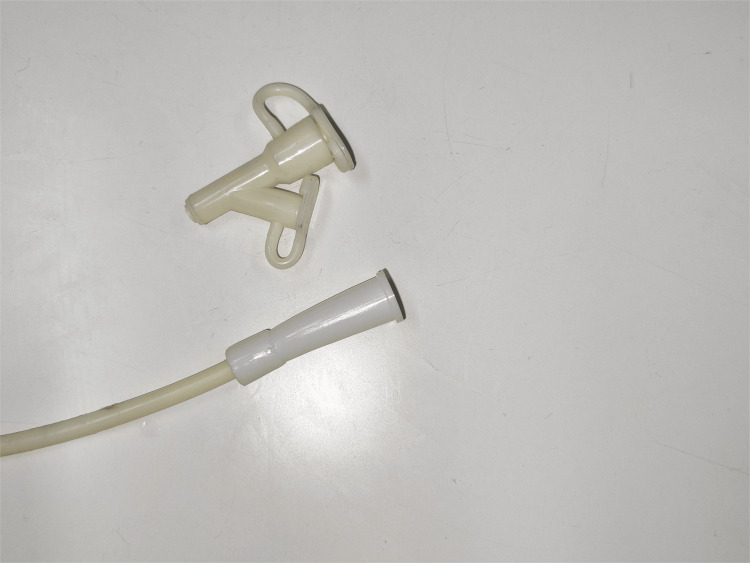
A 12 Fr NGT port is applied over the cut end of the NJT. NGT, nasogastric tube; NJT, nasojejunal tube

**Figure 5:**
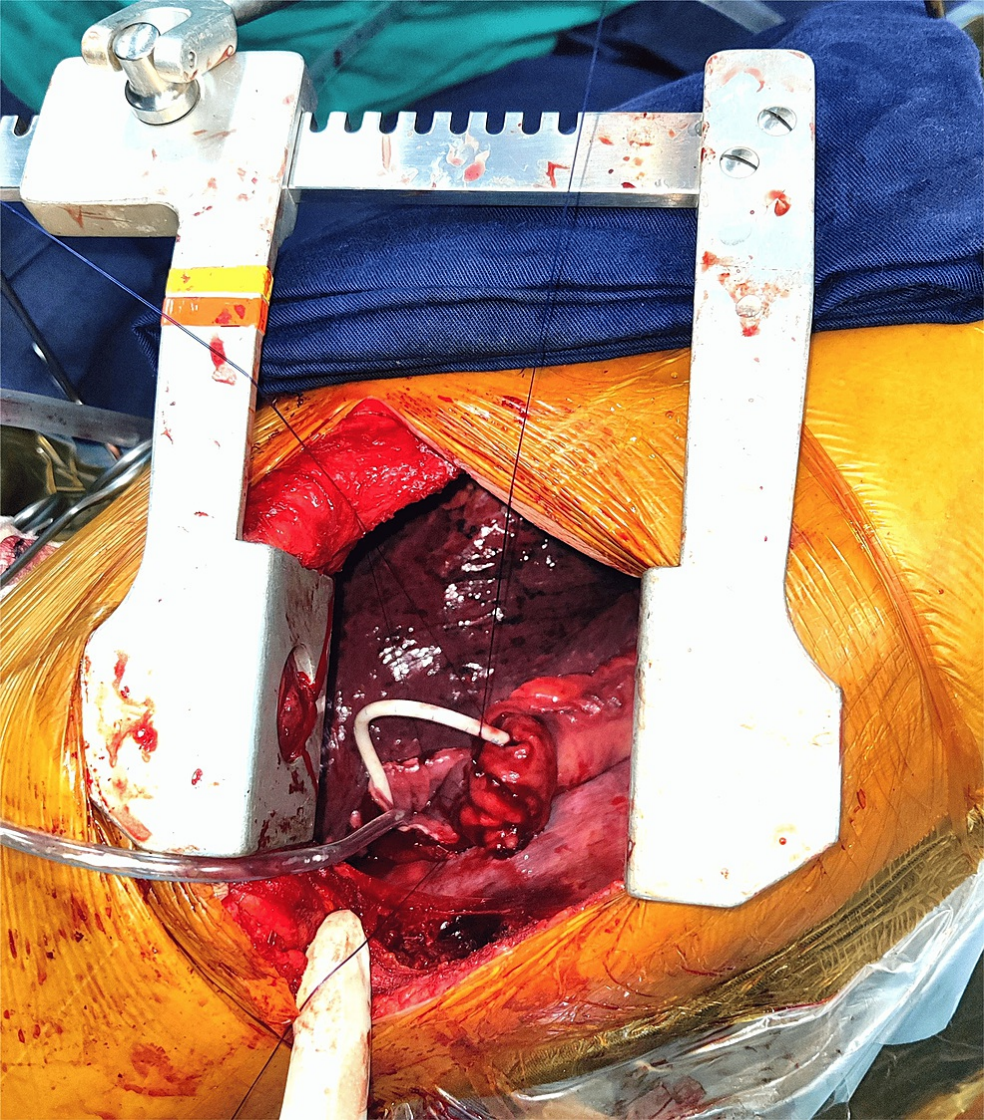
End-to-side esophagogastric anastomosis around the NJT. NJT, nasojejunal tube

Thoracotomy was carried out and the esophagus mobilized to the required level. The partially prepared conduit was delivered into the thoracic cavity. The esophagus was divided proximally using diathermy, taking care not to damage the NJT passing through it. After stabilizing the tube, the guide wire was removed. The port at the end of the NJT was cut using scissors and the cut end of the NJT was sutured in an end-to-end fashion with a 12 Fr nasogastric tube (NGT). The NJT was then pulled into the thoracic cavity until the suture site between the NJT and NGT was visualized. The tubes were then separated. An opening was made on the side of the gastric conduit at the site selected for anastomosis. The NJT was pulled out of it so that it was no longer passing through the bridge of gastric tissue connecting the conduit and the specimen (Figures 1, 2). At this point, an endostapler was used to complete the distal transection, thereby separating the conduit and specimen (Figure 3). The NJT and NGT were again sutured together in an end-to-end fashion and the NGT was pulled by the anesthetist till the sutured junction between the NJT and NGT was out of the nasal cavity. The two tubes were separated and a 12 Fr NGT port was snugly applied onto the cut end of the NJT (Figure 4). A separate NGT was inserted and its distal end was positioned midway between the anastomosis and the pylorus. This kept the conduit decompressed, thereby reducing mechanical forces on the anastomosis. Hand sewn end to side esophagogastric anastomosis was carried out around the NJT (Figure 5).

**Figure 6:**
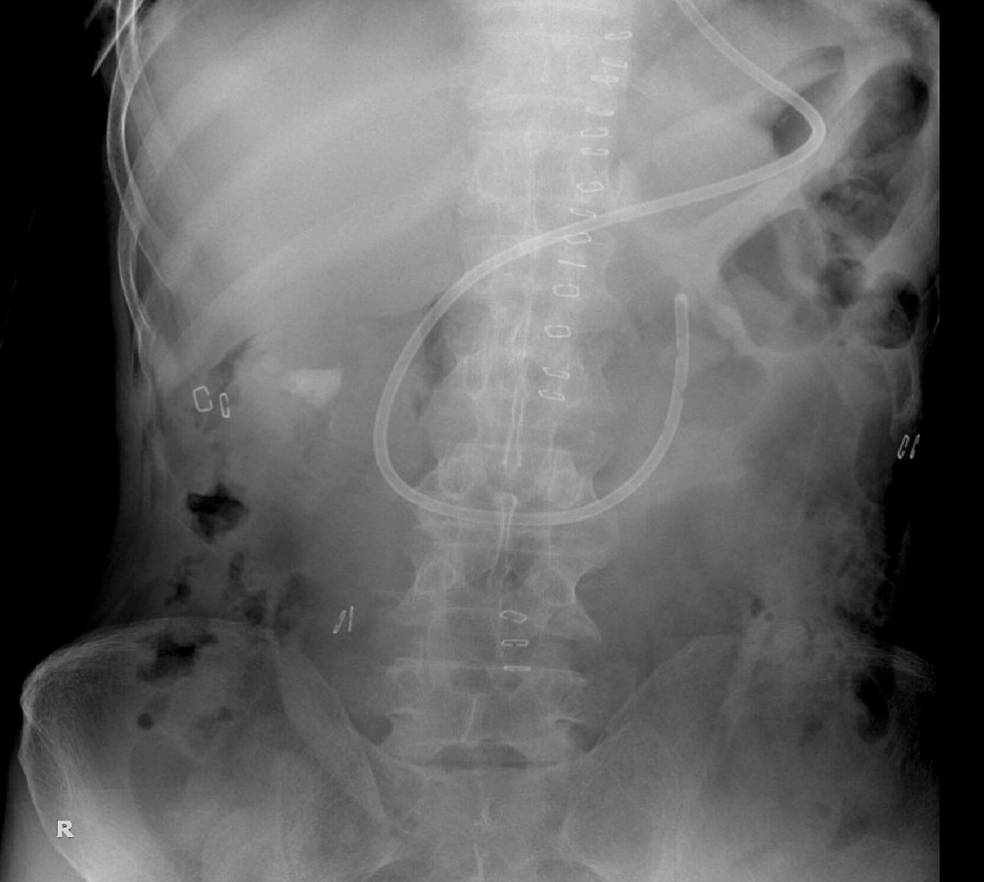
X-ray abdomen confirming the correct position of NJT at the end of the procedure. NJT, nasojejunal tube

At the end of the procedure, a simple abdominal X-ray was carried out to confirm the correct position of the tube (Figure 6). Feeding through NJT was initiated on the first post-operative day (POD). The patient had an uneventful post-operative course and was discharged on POD 6 with instructions to take a blended diet through the tube. On POD 10, NJT was removed and oral diet started. 

## Discussion

Enhanced Recovery after Surgery (ERAS) Society guidelines advocate early enteral feeding; however, the ideal route of administration remains an area of controversy [4]. Early oral feeding may lead to a shorter duration of hospital stay and improve short-term quality of life by minimizing distress associated with feeding tubes and experiences of dry mouth and thirst [3]. However, there is an increased risk of vomiting and aspirational pneumonia with early oral feeding [3]. Patients with esophageal malignancies are typically malnourished on account of reduced oral intake; timely nutritional build-up is, therefore, of paramount importance. It has been shown that the median caloric intake in patients with early oral feeding was suboptimal [6]. This is in contrast to tube feeding, where caloric requirements are met much earlier.

The use of NJT for enteral feeding ensures adequate caloric intake while avoiding feeding jejunostomy-related complications such as peri catheter dermatitis, wound infection, intestinal obstruction, and peritonitis. Possible complications of NJT placement include displacement of the tube, failure of placement, clogging of the tube, and irritation of the nasal cavity [7]. NJT is typically placed endoscopically or under fluoroscopic guidance at the end of the procedure in the Ivor-Lewis esophagectomy. This carries the risk of exposing the anastomosis to mechanical forces generated by the air insufflation and local trauma of the endoscope [8]. Our technique allows the passage of NJT without the use of an endoscope or fluoroscopic guidance.

The limitations of our technique include its incompatibility with stapled anastomosis. The hand-sewn anastomosis is more time-consuming and can prolong the surgery duration. 

## Conclusions

In conclusion, early enteral feeding and adequate caloric intake are of paramount importance in patients undergoing esophagectomy. NJT placement in Ivor-Lewis esophagectomy can be challenging and traditional methods of NJT placement require either endoscopy or fluoroscopic guidance. This carries the risk of exposing the anastomosis to mechanical forces generated by the air insufflation and local trauma of the endoscope. Using our technique, NJT can be easily placed without the use of endoscopy or fluoroscopy and can be utilized for early enteral feeding so that the caloric requirements of the patient may be fulfilled. Limitations of this technique include its incompatibility with stapled anastomosis and prolonged surgery duration if the operator is not experienced with hand-sewn anastomosis. The safety of this technique can be investigated by further studies.
